# Mesenchymal stromal/stem cells for neurological disorders in humans: an evidence-mapped clinical review

**DOI:** 10.3389/fncel.2026.1844360

**Published:** 2026-06-10

**Authors:** Guilherme Lepski, Analía Arévalo

**Affiliations:** 1 Departments of Neurology and Psychiatry, Medical School, São Paulo, Brazil; 2 Laboratory of Experimental Surgery (LIM26), University of São Paulo Medical School, São Paulo, Brazil

**Keywords:** clinical review, mesenchymal stem cell, MSCs, MSCs in neurology, neurological disorders

## Abstract

Mesenchymal stromal/stem cells (MSCs) have been tested clinically across a wide spectrum of neurological disorders, motivated by their immunomodulatory and trophic (“bystander”) mechanisms rather than durable neural replacement. Here, we synthesize human prospective clinical trials that administered MSC products for neurological indications, prioritizing study design/goals, disease stage/severity, cell source/manufacturing, dose/route, detailed clinical assessments, quantified score changes, and adverse events (AEs). Across indications, trials frequently demonstrate feasibility and short-term safety, while efficacy signals are heterogeneous and strongly dependent on disease stage and endpoint selection criteria. The most methodologically rigorous signals with quantified motor outcomes include stereotactic intracerebral implantation of SB623 for chronic motor deficits after traumatic brain injury (TBI). In amyotrophic lateral sclerosis (ALS), randomized evidence supports safety and early slope-based signals in selected subgroups after intrathecal MSC regimens, but durable clinical benefit remains unproven. In hypoxic–ischemic encephalopathy (HIE), controlled data suggest functional improvements in small cohorts, and neonatal studies support feasibility adjunctive to hypothermia. We highlight design features most likely to de-risk efficacy interpretation: adequately powered randomized controlled trials, disease-stage stratification, prespecified clinically meaningful change thresholds, standardized rehabilitation co-interventions, and transparent AE adjudication.

## Introduction

Mesenchymal stromal/stem cells (MSCs) are non-hematopoietic stromal cells typically expanded ex vivo from bone marrow, adipose tissue, or perinatal tissues and characterized by plastic adherence, a consensus surface marker profile, and tri-lineage differentiation capacity under defined conditions (Mehlhorn et al., 2006). Stem cells can be divided into three main groups: (1) embryonic stem cells; (2) fetal stem cells; and (3) adult stem cells. The first type is obtained from the inner layer of the blastocyst and is considered to be pluripotent (able to generate a whole organism or tissues from the three embryonic layers). In 2006, a method was described to isolate these cells from adult tissue, reprogramming them to a more immature state by transfecting certain neurogenic genes and oncogenes (like c-Myc and Klf4; Takahashi and Yamanaka, 2006), the latter being responsible for the major drawback of the strategy: the occurrence of malignant teratoms (Miura et al., 2009). Researchers realized that immaturity/pluripotency is indissociable from oncogenicity. To overcome this major limitation, recent methods have used chemical induction with valproic acid, stem cell transcription factor NANOG, or even CRISPR-Cas9 technology (for an extensive review on these strategies, see Abusalah et al., 2024). Although promising, the production method is still very complex and not widely replicable. The second stem cell type, fetal cells, are obtained from human fetuses from spontaneous or provoked abortions, and are therefore very scarce and not scalable. This type of stem cell was the first to be extensively investigated for neuro-regeneration, but the lack of convincing improvement has led to the search of other stem cell types (Barker et al., 2026). The last type, adult stem cells, can be obtained from adult tissue, and can therefore be obtained from the target patient, thus avoiding rejection or the need for immunosuppression (Mariano et al., 2015). Isolation and expansion of these cells is relatively simple, and some sources like blood-derived cells have been used clinically for a long time in the context of bone marrow replacement following aggressive chemotherapy, which has an unquestionably favorable bio-safety profile. Nevertheless, their neurogenic potential is naturally limited compared with the other types of stem cells mentioned (Lepski, 2012; Lepski et al., 2010a, 2010b). Based on this observation, it is assumed that in neurological conditions, the dominant therapeutic hypothesis is paracrine immunomodulation and trophic support—including downregulation of innate/adaptive inflammatory cascades, modulation of microglia/macrophage phenotypes, secretion of neurotrophic and angiogenic factors, endothelial stabilization, and extracellular vesicle signaling—rather than stable neuronal replacement (Mariano et al., 2015). Clinical translation has therefore tested diverse products (autologous vs. allogeneic; minimally manipulated vs. culture-expanded; “primed” or engineered MSCs) and delivery strategies (intravenous, intra-arterial, intrathecal, intraparenchymal), each with distinct biodistribution and risk profiles, as well as interpretability of efficacy endpoints.

The clinical literature has expanded quickly but remains heterogeneous, and for some indications, trial governance and reporting quality have been questioned, as occurred in the case of a recent prominent autism spectrum disorder (ASD) UC-MSC publication, which was later retracted (Retraction, 2021).

Our goal with this review is to provide a much-needed overview of the quality and consistency of the clinical trials published so far, addressing serious adverse neurological conditions and objectively assessing the reported outcomes as they correlate with each stem cell source, isolation method, and transplantation technique. Finally, we highlight the limitations and future directions of this important line of research.

## Methods

### Search strategy and study selection

A structured search was performed primarily in PubMed, Socpus and Web of Knowledge restricted to the last 12 years, using combinations of: *mesenchymal*, *stromal*, *stem cell*, *MSC*, *clinical trial*, *randomized*, *placebo*, and disease-specific terms related to severe neurological conditions (stroke, SCI, MS, ALS, TBI, cerebral palsy, Alzheimer). We prioritized human interventional trials (phase I–III; any language), and also screened ClinicalTrials.gov entries when a publication explicitly reported the NCT identifier or when registry details clarified dosing/route. Representative randomized clinical trials (RCTs) and well-characterized phase I/II trials were selected for detailed extraction.

### Data extraction fields

For each trial we extracted: (a) N, (b) design/primary goals, (c) diagnosis + severity/stage, (d) MSC source, (e) manufacturing/isolation + dose/route/schedule, (f) assessments, and (g) results.

### Further MSC specifications to consider

Across trials, we consider the source of MSCs (bone marrow aspirate/adipose/umbilical cord tissue or cord blood), plastic adherence, ex vivo expansion phase (often passage ~2–5), detailed information on bio-security or GMP standards (sterility, endotoxin levels), and phenotyping consistent with International Society for Cell and Gene Therapy (ISCT) criteria (typically: CD73/CD90/CD105 positive; CD45/CD34 negative; tri-lineage differentiation reported). This strategy prevented the inclusion of unprocessed mesenchymal tissue. We prioritized studies with (i) clearly described MSC source and manufacturing (at minimum: tissue source and culture expansion methods), (ii) defined dose and route of administration, (iii) standardized clinical assessments with baseline and follow-up values (or change from baseline), and (iv) explicit adverse event (AE) reporting. When full-score trajectories or AE tables were not available, we explicitly state “not reported/not accessible”.

Results are organized by disease category. Supplementary Table S1 summarizes trial-level details, including additional registries and programs.

## Results

### Traumatic brain injury

Traumatic brain injury (TBI) represents the 7th cause of disability worldwide, with annual incidence of 27,000 cases per 100,000 inhabitants, what is responsible for 8,000 DALYs (disability-adjusted life years; Feigin et al., 2019). The strongest controlled evidence with sham surgery and prespecified motor impairment endpoints comes from the STEMTRA program, which used SB623, an allogeneic, culture-expanded, modified bone marrow–derived MSC product delivered via stereotactic intracerebral implantation (Kawabori et al., 2021; Okonkwo et al., 2024). In the randomized, double-blind, sham-controlled trial with 24- and 48-week *post hoc* analyses, participants were ≥12 months post moderate-to-severe TBI with chronic motor deficits. SB623 was implanted stereotactically into the peritrauma brain tissue responsible for motor deficits, as identified by MRI; sham controls underwent a similar stereotactic burr-hole procedure without dural penetration (Okonkwo et al., 2024).

The primary endpoint was change from baseline on the Fugl–Meyer Motor Scale (FMMS) at 24 weeks. Baseline FMMS means were approximately 52 across arms (SB623 pooled 52.2 ± 19.3; sham 52.3 ± 15.1). At 24 weeks, least-squares mean FMMS change from baseline was +8.3 (SE 1.4) for pooled SB623 versus +2.3 (SE 2.5) for sham (*p* = 0.04). At 48 weeks, FMMS change from baseline was +7.5 (SE 1.3) for pooled SB623 versus +4.1 (SE 2.2) for controls (between-group *p* = 0.20; Okonkwo et al., 2024).

Throughout the 48 weeks, treatment-emergent serious adverse events occurred in 4/46 (8.7%) SB623-treated participants versus 3/15 (20%) controls; most were determined to be unrelated to the cells or unlikely related to surgery and were largely resolved (Okonkwo et al., 2024).

### Spinal cord injury

Spinal cord injury (SCI) is the 6th global cause of disability worldwide and affects 935 persons per 100,000 inhabitants/year. Because it affects mostly young patients, it represents 9,500 DALYs (Feigin et al., 2019). The lack of neurogenesis in the adult spinal cord and the presence of multiple molecules that inhibit axo-dendritic growth make the use of regenerative strategies in SCI especially challenging (Mili and Choudhary, 2024). SCI trials span intrathecal delivery of autologous adipose-derived MSCs (AD-MSCs), autologous bone marrow MSCs, and combined approaches. The CELLTOP phase I trial delivered intrathecal autologous culture-expanded AD-MSCs to 10 individuals with traumatic SCI (AIS A/B at injury) to evaluate feasibility and safety (Bydon et al., 2024). The most common adverse events were headache and musculoskeletal pain (8/10), and no serious adverse events were observed. At final follow-up, seven participants improved in AIS grade from the time of injection (Bydon et al., 2024).

Earlier intrathecal AD-MSC series report feasibility in predominantly AIS A chronic SCI cohorts (Hur et al., 2016).

### Hypoxic–ischemic encephalopathy

Hypoxic–ischemic encephalopathy (HIE) is a form of global brain injury caused by insufficient oxygen (hypoxia) and/or reduced cerebral blood flow (ischemia). It can occur perinatally (around birth) after events such as placental abruption, umbilical cord compromise, severe maternal hypotension, or prolonged difficult delivery, but can also occur in older children and adults after cardiac arrest, shock, or severe respiratory failure. The incidence of perinatal HIE is higher in undeveloped countries (ranging from 1.5 to 20 per 1,000 live births; Chakkarapani et al., 2025). Adult HIE, on the other hand, is more difficult to estimate; some studies indicate a global incidence of 55 per 100,000 person-years (Yan et al., 2020); most importantly, roughly 1.5% remain in a persistent vegetative/unresponsive wakefulness state (Sandroni et al., 2021), for which no effective treatment exists. It is associated with a very high likelihood of lasting neurodevelopmental or neurological disabilities, even when optimal care is available.

Controlled clinical data exist for MSC transplantation in non-neonatal hypoxic–ischemic encephalopathy (HIE). In a controlled clinical study (*n* = 22; 12 treated vs. 10 controls), Xie et al. evaluated human umbilical cord-derived MSCs and assessed neurologic impairment and function using NIHSS (lower is better), Barthel Index (higher is better), MMSE (higher is better), HAMA-24 and HAMD-14 (lower is better), and UPDRS (lower is better) at baseline and at days 14, 90, and 180. Both groups improved, but the MSC group showed larger gains in activities of daily living and cognition, with greater Barthel Index improvement at day 14 (+13.64 ± 5.52 vs. +6.50 ± 5.30; *p* = 0.011) and day 90 (+15.91 ± 8.61 vs. +8.00 ± 5.87; *p* = 0.025), and consistently larger MMSE increases at day 14 (+5.00 ± 5.17 vs. +0.60 ± 1.78; *p* = 0.015), day 90 (+4.83 ± 4.11 vs. +0.80 ± 1.23; *p* = 0.005), and day 180 (+4.92 ± 4.81 vs. +0.90 ± 1.66; *p* = 0.029). UPDRS improvements favored MSCs at day 90 (−10.40 ± 6.19 vs. −5.10 ± 4.33; *p* = 0.033) and day 180 (−11.10 ± 6.47 vs. −5.40 ± 3.37; *p* = 0.017), while NIHSS changes showed a non-significant trend toward larger neurologic deficit reduction by day 180 (−5.64 ± 3.01 vs. −3.60 ± 1.96; *p* = 0.058). The authors reported no significant adverse effects during the 180 days of follow-up in the treated cohort (Xie et al., 2016).

In neonatal HIE, an exploratory multicenter randomized parallel-group phase I/II trial in Japan compared whole-body therapeutic hypothermia alone versus hypothermia plus an off-the-shelf, allogeneic bone marrow–derived human MSC product (TEMCELL HS Inj.; “Temcell”; Wada et al., 2025). Fourteen neonates were randomized (7 per group); one infant in the Temcell arm was excluded from efficacy evaluation due to protocol deviation (full analysis set for efficacy: 6 Temcell vs. 7 control). Stratified block randomization used Thompson score ≥11 vs. <11, and most infants were Sarnat stage 2 with one stage 3 infant in each arm. The first Temcell dose was administered within 12–36 h after birth once core temperature was stabilized at 33–34 °C under hypothermia; Temcell was infused intravenously over 10 min at 2.0 × 10^6^ cells/kg (diluted in 18 mL saline per bag), twice weekly for 4 weeks (≥3 days between doses). Five of six Temcell-treated infants received steroid premedication to prevent allergic reactions. The primary endpoint was treatment response defined as an overall developmental quotient (DQ) ≥ 85 at 18 months, assessed centrally and blinded using the Kyoto Scale of Psychological Development 2001 (KSPD), which correlates with BSID-III. Treatment response occurred in 4/6 (66.7%) infants in the Temcell + hypothermia group and 4/7 (57.1%) in the hypothermia-only group; in the blinded independent case review committee’s final overall assessment, response was recorded in 4/6 (66.7%) vs. 5/7 (71.4%), respectively. Mean overall DQ at 12 and 18 months was 98.7 and 91.0 in the Temcell arm versus 86.0 and 88.4 in controls. Secondary endpoints included BSID-III; MRI injury grade at 10 days and 18 months; GMFCS at 12 and 18 months; summary Thompson scores on postnatal days 1–3 (means: 11.3/11.8/10.5 Temcell vs. 12.0/11.4/8.0 control); and clinical events (seizures, gastrostomy, tracheostomy/ventilation, inotropes). At 18 months, MRI was grade 0 (normal) in 4 Temcell infants, grade 2A in 1, and missing in 1; controls had grade 0 in 6 and grade 3 in 1. GMFCS at 18 months was level I in five Temcell infants (missing in 1) and level I in six controls with one level V. Safety profiles were comparable: adverse events occurred in all infants (65 events Temcell vs. 60 control), with no deaths and no severe events; three moderate events (adrenal insufficiency, diabetes insipidus, tibial fracture) occurred in two Temcell infants. Adverse drug reactions (all mild, resolved) were reported in three Temcell infants (42.9%): supraventricular extrasystoles, hypotension, and cholestasis (each 14.3%). Common AEs included diaper rash and infantile eczema in the Temcell arm, and diaper rash and laryngitis in controls (Wada et al., 2025).

### Amyotrophic lateral sclerosis

Amyotrophic lateral sclerosis (ALS) is a progressive neurodegenerative disease of upper and lower motor neurons, leading to relentlessly worsening muscle weakness, atrophy, spasticity, dysarthria, dysphagia, and ultimately respiratory failure (Feldman et al., 2022). Worldwide, ALS is uncommon but not rare. A large systematic review reports substantial geographic variation in point prevalence, ranging roughly from ~1.6 per 100,000 in some countries to ~11.8 per 100,000 in others (Wolfson et al., 2023). ALS severity is due to its irreversible and progressive course, during which many patients develop bulbar dysfunction (speech/swallowing) and respiratory muscle weakness, requiring ventilatory support (Feldman et al., 2022). It also carries a major burden of disability and caregiver dependence, and a meaningful fraction of patients develop cognitive/behavioral impairments. Prognosis is generally poor, and typical survival is on the order of ~2–5 years from symptom onset (Chiò et al., 2009). MSC strategies for ALS include repeated intrathecal autologous bone marrow MSC dosing and MSCs induced to secrete neurotrophic factors (MSC-NTF). In a randomized phase II trial, 64 participants received riluzole alone (*n* = 31) or riluzole plus two repeated intrathecal injections of autologous bone marrow-derived MSCs (*n* = 33). The trial reported acceptable safety and a possible clinical benefit lasting at least 6 months, with longitudinal ALSFRS-R analyses and biomarker shifts consistent with immunomodulation (Oh et al., 2018).

In a randomized placebo-controlled phase II trial of MSC-NTF (NurOwn), 48 participants were randomized 3:1 (MSC-NTF *n* = 36; placebo *n* = 12). Baseline ALSFRS-R was similar across arms (38.1 ± 3.6 vs. 38.6 ± 3.85). Safety endpoints were met with no treatment-related serious adverse events; transient procedure-related adverse events were common. Prespecified analyses suggested early slope improvements in rapid progressor subgroups, supporting the rationale for responder enrichment strategies (Berry et al., 2019).

### Multiple sclerosis

Multiple sclerosis (MS) is a chronic immune-mediated inflammatory disease of the central nervous system characterized by demyelination and neuroaxonal injury, leading to relapses and/or progressive disability. Multiple Sclerosis is one of the most common causes of non-traumatic neurological disability in young adults (Jakimovski et al., 2023). Worldwide, Global Burden of Disease–based analyses report about ~1.8 million prevalent cases in 2019 (and show rising burden over time; Walton et al., 2020). Multiple Sclerosis is severe because it frequently produces long-term motor, sensory, visual, cerebellar, cognitive, and fatigue-related impairment, and many patients transition from relapsing disease to secondary progressive disability despite therapy (Jakimovski et al., 2023). Prognosis is highly heterogeneous—ranging from relatively mild courses to rapidly progressive disability—but overall mortality is increased compared with the general population and life expectancy is meaningfully reduced, on average (often by several years), largely due to complications associated with advanced disability and comorbid diseases (Scalfari et al., 2013).

MSC trials for MS include randomized, double-blind, placebo-controlled crossover studies using intravenous autologous bone marrow MSCs with MRI gadolinium-enhancing lesions and disability scales (EDSS) as key endpoints (Llufriu et al., 2014). The largest study to date, MESEMS, was a multicenter randomized, double-blind, placebo-controlled phase II crossover trial conducted at 15 sites in nine countries enrolling patients aged 18–50 years with active relapsing–remitting or progressive MS (disease duration 2–15 years; EDSS 2.5–6.5). Participants were assigned 1:1 to receive a single intravenous infusion of autologous bone marrow–derived MSCs followed by placebo at week 24, or placebo followed by MSCs at week 24, with follow-up to week 48. The primary efficacy endpoint was the cumulative number of gadolinium-enhancing lesions counted over weeks 4, 12, and 24. MSC treatment did not reduce the total number of gadolinium-enhancing lesions from baseline to week 24 compared with placebo (rate ratio 0.94, 95% CI 0.58–1.50; *p* = 0.78). The authors concluded that bone marrow–derived MSC treatment was safe and well tolerated but did not show an effect on this MRI surrogate marker of acute inflammation at week 24; they further emphasized that future trials should focus on endpoints related to tissue repair rather than acute inflammatory activity (Uccelli et al., 2021).

### Parkinson’s disease

Parkinson’s disease (PD) is a progressive neurodegenerative syndrome classically caused by degeneration of dopaminergic neurons in the substantia nigra, with widespread *α*-synuclein pathology producing bradykinesia plus tremor and/or rigidity, as well as some prominent non-motor symptoms (autonomic dysfunction, sleep disorders, mood/cognition) and gait/postural instability (Bloem et al., 2021). Globally, PD is one of the fastest growing neurological disorders; a 2021 GBD-based analysis estimated about 11.77 million people living with PD in 2021 (up from ~3.15 million in 1990), highlighting a steep rise largely driven by population aging (Li et al., 2025). Due to its chronic and progressive course, PD may cause substantial disability from falls, dysphagia/aspiration risk, frailty, and cognitive decline in a sizable subset of patients; prognosis is heterogeneous (some live many years with mild disability, others progress quickly), but long-term morbidity is typically high, even with optimized dopaminergic therapy and supportive care (Bloem et al., 2021).

Prospective clinical trials of cell transplantation for PD have a long arc. Early open-label fetal ventral mesencephalic (VM) grafting in the 1990s showed proof-of-concept (including PET evidence of dopaminergic function), which led to pivotal sham-surgery–controlled trials in the early 2000s. The NEJM trial of embryonic dopamine neuron implantation and the Annals of Neurology double-blind fetal nigral transplantation trial produced mixed clinical benefit and highlighted important complications—particularly graft-induced dyskinesias in a substantial fraction of transplanted patients—triggering a field-wide reassessment of patient selection, tissue preparation, surgical targeting, immunosuppression, and endpoints (Freed et al., 2001; Olanow et al., 2003). Those lessons directly informed the European TRANSEURO effort, which was designed as an open-label, carefully standardized framework intended to de-risk the variables that undermined earlier trials and to pave the way toward scalable stem-cell–derived dopaminergic neuron products. The latest major report from TransEuro described grafting human fetal ventral mesencephalic tissue in 11 patients at two centers using a harmonized tissue protocol but different implantation devices; at 3 years, there was no overall clinical effect on the primary endpoint, no major graft-induced dyskinesias, and outcomes differed by device and/or site, reinforcing both the feasibility limits of fetal tissue and the rationale for standardized stem-cell sources (Barker et al., 2026).

In PD, most MSC-based studies are early-phase and safety-oriented. A phase I dose-escalation study administered a single intravenous infusion of allogeneic bone marrow-derived MSCs (1–10 × 10^6^ cells/kg) to 20 individuals with idiopathic PD. The study reported no serious adverse reactions related to infusion and tracked treatment-emergent events; clinical efficacy endpoints were exploratory and should be interpreted as hypothesis-generating (Schiess et al., 2021).

### Multiple system atrophy

Multiple system atrophy (MSA) is a sporadic, adult-onset, rapidly progressive neurodegenerative disorder characterized by varying combinations of autonomic failure (neurogenic orthostatic hypotension, urinary dysfunction), parkinsonism (often with poor levodopa response), and cerebellar ataxia, with pathology defined by *α*-synuclein–positive glial cytoplasmic inclusions (Krismer et al., 2024; Mandrekar et al., 2014). Epidemiologically, MSA is rare and estimates vary by region and case ascertainment; a recent literature review reports crude prevalence ranging from ~0.5 to 17 per 100,000, with many studies clustering around ~5–10 per 100,000 (Kaplan, 2024). MSA is considered severe because it progresses quickly to multisystem disability, with prominent autonomic complications, falls, dysphagia/aspiration risk, and frequent need for mobility aids and institutional support. Prognosis is poor: cohort studies show median survival in the ~8–9-year range with substantial individual variability, with earlier and more severe autonomic dysfunction often associated with worse outcomes (Figueroa et al., 2020).

MSA programs have frequently used endovascular delivery to increase CNS exposure, but this strategy introduces route-specific vascular risks. In an open-label, single-center phase I dose-escalation trial (*n* = 9) in probable MSA-cerebellar type (MSA-C) with relatively early disease (total UMSARS 30–50; disease duration <5 years), Chung et al. administered a single intra-arterial infusion of autologous bone marrow–derived MSCs (BM-MSCs) at 3.0 × 10^5^, 6.0 × 10^5^, or 9.0 × 10^5^ cells/kg, delivered via femoral access with a catheter positioned sequentially in the cervical segments of both internal carotid arteries and the proximal dominant vertebral artery (1/4 of the cell volume into each ICA and 1/2 into the dominant VA). Stem cells were isolated from bone marrow aspirate using Ficoll density-gradient centrifugation, culture-expanded under GMP, and characterized by flow cytometry (CD34−/CD45−, CD29+/CD44+/CD73+/CD90+/CD105+). Safety monitoring included serial neurologic examinations and brain MRI 1 day after infusion to detect procedure-related ischemia. Fifteen adverse events were recorded in 5/9 participants; a single moderate suspected unexpected serious adverse reaction compatible with transient blood–brain barrier disruption (leptomeningeal enhancement on MRI) occurred in one low-dose participant and resolved without new deficits; no diffusion-weighted MRI ischemic lesions were observed in any participant. Other AEs were predominantly mild and included headache (1/9), photopsia (1/9), constipation (2/9), abdominal distension (1/9), increased blood pressure (1/9), weight increase (1/9), herpes zoster (1/9), urinary tract infection (1/9; serious), fall (1/9), rib fracture (1/9), and anxiety (1/9). Exploratory efficacy analyses used UMSARS at baseline (V1, −1 month), 1 month (V7), and 3 months (V8) and suggested numerically slower worsening in the pooled medium/high-dose versus low-dose cohort, although these differences were not statistically significant (group × time *p* = 0.131 for UMSARS part II; *p* = 0.096 for total). Estimated means (SE) illustrate the divergence by 3 months: total UMSARS increased from 43.00 (2.79) to 55.33 (5.14) in the low-dose group versus 37.67 (1.97) to 40.83 (3.64) in the medium/high-dose group; UMSARS part II increased from 20.33 (1.73) to 25.33 (2.39) versus 18.00 (1.22) to 18.33 (1.69), respectively (Chung et al., 2021). In a randomized placebo-controlled trial (*n* = 33) in probable MSA-C with baseline total UMSARS 30–50, Lee et al. (2012) administered autologous MSCs (4 × 10^7^ cells per injection) via combined intra-arterial and intravenous routes and reported a significantly smaller increase in total UMSARS and UMSARS part II over 360 days compared with placebo in mixed-model analyses (interaction *p* = 0.047 and *p* = 0.008, respectively). The placebo group showed more extensive declines in cerebral glucose metabolism and gray matter density, with greater deterioration in frontal cognition, whereas no serious adverse effects were directly attributed to MSCs; however, intra-arterial infusion was associated with small ischemic MRI lesions, underscoring the need to balance potential disease-modifying signals against endovascular risk (Lee et al., 2012).

### Cerebral palsy

Cerebral palsy (CP) is a group of permanent movement and posture disorders causing activity limitation, attributed to a non-progressive disturbance/lesion in the developing fetal or infant brain, and commonly accompanied by comorbid impairments (e.g., epilepsy, cognition/communication, vision/hearing) and secondary musculoskeletal complications (Sadowska et al., 2020). Epidemiologically, CP is the most common childhood-onset physical disability; a global systematic analysis reports that in high-income countries, CP birth prevalence has declined to about ~1.6 per 1,000 live births, while available data from low- and middle-income countries suggest substantially higher prevalence, though estimates are more variable due to surveillance limitations (McIntyre et al., 2022). Cerebral palsy is a lifelong condition that can involve significant motor impairment (sometimes with feeding/swallowing dysfunction, scoliosis/contractures, pain, and epilepsy), leading to high care needs and reduced participation; prognosis is highly heterogeneous—many individuals with milder motor involvement have long survival, but mortality is higher than the general population, especially in severe disability. Respiratory illness/aspiration-related complications are a leading contributor to morbidity and mortality (Marpole et al., 2020).

Randomized controlled MSC trials in cerebral palsy (CP) have generally used GMFM-based motor outcomes and commonly combine cell therapy with background rehabilitation. In a placebo-controlled, single-blind trial of human umbilical cord blood–derived MSCs (hUCB-MSCs; *n* = 54; 27 MSC vs. 27 placebo), all children received basic rehabilitation and the intervention arm received four fixed-dose intravenous infusions of 5 × 10^7^ hUCB-MSCs, whereas controls received saline (Huang et al., 2018). Baseline gross motor function was similar between groups (GMFM-88 total score proportion 84.99 ± 0.85 vs. 85.03 ± 0.76). Improvement was quantified as change from baseline in the GMFM-88 total score proportion, which was greater with hUCB-MSC therapy at every assessed time point (3/6/12/24 months: 4.59 ± 0.26/7.62 ± 0.47/10.27 ± 0.57/12.66 ± 0.66 vs. 1.74 ± 0.39/2.96 ± 0.32/4.75 ± 0.28/4.81 ± 0.39; *p* < 0.05 as reported). Comprehensive functional assessment (CFA) also favored hUCB-MSCs, with greater total score gains over 3/6/12/24 months (7.2 ± 0.73/12.0 ± 0.97/18.9 ± 1.15/25.0 ± 1.23 vs. 2.9 ± 0.33/5.5 ± 0.52/8.07 ± 0.54/10.6 ± 0.65; *p* < 0.05 as reported). Safety monitoring did not identify serious adverse events; non-serious events were common in both arms and were predominantly mild/moderate (e.g., upper respiratory tract infection and diarrhea; Huang et al., 2018). In a randomized, double-blind, placebo-controlled trial of human umbilical cord–derived MSCs (hUC-MSCs) administered intravenously in conjunction with rehabilitation (39 treated; 19 MSC vs. 20 placebo), Gu et al. assessed change-from-baseline in ADL, CFA, and GMFM-88 at 1, 3, 6, and 12 months after the last dose (Gu et al., 2020). Between-group differences emerged later for GMFM, with greater GMFM improvement at 6 months (59.00 ± 8.947 vs. 28.90 ± 8.807; *p* = 0.037) and 12 months (64.526 ± 9.600 vs. 36.800 ± 8.802; *p* = 0.045), while earlier differences at 3 months trended but were not significant (32.053 ± 5.028 vs. 15.700 ± 6.746; *p* = 0.062). Functional gains in ADL and CFA were evident earlier (ADL: 3 months 12.447 ± 2.156 vs. 5.975 ± 1.782, *p* = 0.013; 6 months 21.053 ± 2.860 vs. 10.125 ± 2.250, *p* = 0.003; 12 months 22.974 ± 2.936 vs. 12.775 ± 2.465, *p* = 0.009; CFA: 3 months 17.947 ± 3.317 vs. 7.425 ± 2.754, *p* = 0.021; 6 months 23.790 ± 3.774 vs. 11.925 ± 3.253, *p* = 0.026). Adverse events were prospectively collected for 12 months; no serious adverse events were observed, and AE incidence did not differ significantly between groups, with upper respiratory infection reported most frequently; in three cases, transient low-grade fever within 24 h of infusion was considered to be related to hUC-MSC transplantation (Gu et al., 2020).

### Autism spectrum disorder

Autism spectrum disorder (ASD) is a lifelong neurodevelopmental condition characterized by persistent differences/difficulties in social communication and interaction together with restricted, repetitive patterns of behavior/interests or sensory features, with marked heterogeneity in language, cognition, adaptive function, and comorbidities (e.g., ADHD, anxiety, epilepsy, intellectual disability). Recent global estimates from the 2021 Global Burden of Disease (GBD) Study suggest that approximately 1 in 127 people worldwide were autistic in 2021 (with burden persisting across the lifespan), while a large systematic review similarly supports a global prevalence on the order of ~1% of children, though estimates vary substantially by region and methodology (Santomauro et al., 2024; Zeidan et al., 2022). As it may entail major, enduring impairments in adaptive functioning, communication, educational attainment, independent living, and social participation, ASD can be considered a severe neurologic/developmental condition; also, it may co-occur with medical and psychiatric conditions that can compound disability. Long-term prognosis is heterogeneous—some individuals achieve good functional independence, while many require ongoing support; reviews of adult outcomes report that a substantial proportion of autistic adults have high levels of dependence and low rates of independent living and stable employment, especially when intellectual disability is present (Eigsti, 2024). Mortality risk is also elevated compared with the general population, particularly among autistic people with intellectual disability, underscoring the clinical importance of lifelong medical care and support systems (O'Nions et al., 2023).

MSC studies for ASD remain early phase and require careful attention to trial governance and interpretation. Sun et al. (2020) reported an open-label phase I dose-escalation study in 12 children with ASD (ages 4–9 years) who received one, two, or three intravenous infusions of a third-party allogeneic human cord tissue MSC product at 2 × 10^6^ cells/kg given at 2-month intervals (Sun et al., 2020). Efficacy endpoints were analyzed descriptively (no hypothesis testing and no control arm). Three ASD-relevant outcome measures were prespecified: the Vineland Adaptive Behavior Scales, Third Edition (VABS-3) Socialization subscale (clinically significant improvement defined as ≥+3 points), the Pervasive Developmental Disorder Behavior Inventory (PDDBI) Autism Composite (clinically significant improvement defined as ≤−5 points), and the Clinician Global Impression of Improvement (CGI-I) scale. By these prespecified criteria, 6/12 (50%) participants improved on ≥2/3 measures, with 4/12 improving on all three measures; improvements were distributed across dose cohorts (two each in the 1-dose, 2-dose, and 3-dose groups). Safety was favorable: aside from agitation associated with IV placement/infusion, no acute infusion toxicity was reported. However, 5/12 participants developed *de novo* class I anti-HLA antibodies (associated with a specific cell lot and/or partial donor–recipient HLA matching), which were clinically silent during follow-up; behavioral/psychiatric adverse events were noted in several children but were generally self-limited (Sun et al., 2020). These findings support feasibility and short-term tolerability, while underscoring that any apparent symptom improvements require confirmation in placebo-controlled trials.

## Discussion

Across neurological disorders, MSC trials most consistently demonstrate feasibility and acceptable short-term safety in controlled settings, whereas efficacy signals are heterogeneous and highly sensitive to study design, endpoint choice, disease stage, and co-interventions. This heterogeneity is not surprising: most neurological disorders targeted in the MSC literature are biologically and clinically heterogeneous, many are progressive, and several exhibit distinct time-courses (e.g., acute/subacute recovery dynamics after TBI or SCI versus chronic neurodegeneration in ALS, PD, MS, and MSA). When trials include participants with broad severity ranges, variable disease durations, and mixed phenotypes, any true treatment effect is likely diluted by competing trajectories (spontaneous recovery, rehabilitation response, and variable natural history). Moreover, for some serious neurological conditions, first-class evidence is difficult to come by, due to the conditions’ rarity (Meijer et al., 2021) or lack of disease-specific animal models (Choudhary, 2025). Therefore, future studies should prioritize participant homogeneity through stringent inclusion criteria and prespecified stratification variables (time from injury/onset, baseline disability, biomarker-defined subtypes, and standardized concomitant rehabilitation).

A second major challenge is product heterogeneity: “MSC” encompasses different tissue sources (bone marrow, adipose, cord tissue/cord blood), donor statuses (autologous vs. allogeneic), culture expansion conditions, passage number, cryopreservation methods, post-thaw viability, and potency testing, all of which can influence secretome composition and immunomodulatory behavior. Even when flow-cytometric identity approximates ISCT criteria, functional potency assays are inconsistently reported, complicating cross-trial comparability. Standardization and large-scale manufacturing under GMP—particularly for multicenter trials—remain difficult, and batch-to-batch variability can be amplified by donor effects, media supplements, and cryopreservation workflows. These translational constraints intersect with regulatory barriers that differ across jurisdictions, particularly for manipulated products, repeat dosing schedules, and invasive delivery routes.

Mechanistic uncertainties further complicate endpoint selection. For systemic (IV) delivery, blood–brain barrier (BBB) penetration is limited and biodistribution is dominated by first-pass pulmonary sequestration; therefore, clinical effects—if present—may reflect peripheral immune modulation, neurovascular signaling, or altered systemic inflammation rather than direct CNS engraftment. For intrathecal delivery, CSF exposure is higher but still does not guarantee parenchymal penetration. For intra-arterial and intraparenchymal approaches, target engagement is more plausible, but procedure-related risks rise. Across routes, the key biological questions are cell survival, engraftment, and persistence: most MSCs are thought to have limited long-term survival after administration, suggesting that durable benefit (if observed) may arise from transient paracrine signaling rather than stable integration; this further reinforces the need for biomarkers of target engagement (e.g., inflammatory signatures, neurofilament dynamics, imaging correlates) and dosing schedules that match expected pharmacodynamics.

The SB623 TBI program illustrates how rigorous design can clarify interpretation in a field vulnerable to placebo and rehabilitation effects. In chronic TBI with stable deficits, stereotactic intraparenchymal implantation with sham surgery and prespecified motor outcomes yielded a statistically significant between-group difference at 24 weeks, with attenuation at 48 weeks, emphasizing the importance of long-term follow-up and clinically meaningful thresholds rather than single time-point significance (Okonkwo et al., 2024). In SCI, AIS conversion and ASIA motor/sensory scores provide interpretable anchors, but small cohorts, variable injury chronicity, and inconsistent rehabilitation dosing limit cross-trial comparability (Bydon et al., 2024; Hur et al., 2016). In non-neonatal HIE, functional and cognitive gains on Barthel Index and MMSE were larger in the MSC arm, but the cohort was small and the phenotype label “HIE” may encompass diverse etiologies and lesion patterns (Xie et al., 2016). In neonatal HIE, the Temcell + hypothermia study underscores the feasibility of adjunctive MSC treatment, yet the small sample and steroid premedication in most treated infants highlight how co-interventions should be standardized or explicitly modeled, as they can confound the interpretation of efficacy (Wada et al., 2025).

In progressive neurodegenerative diseases, trial design must respect both heterogeneity and the expected pace of decline. ALS trials suggest that stratification by progression rate may be essential to detect transient slope-based signals, and the field is moving toward responder enrichment and biomarker-anchored endpoints rather than broad, unstratified cohorts (Berry et al., 2019; Oh et al., 2018). In MS, the MESEMS trial was safe and well tolerated but did not reduce acute inflammatory MRI activity, reinforcing that MSCs may be better tested against endpoints reflecting tissue repair, network reorganization or disability stabilization, rather than short-term inflammation alone (Uccelli et al., 2021). In MSA, intra-arterial trials suggest possible slowing signals but also reveal vascular and BBB-related imaging risks that must be weighed against uncertain efficacy (Chung et al., 2021; Lee et al., 2012).

Immune compatibility is a third translational axis. Although MSCs are often described as being immune-evasive, allogeneic dosing—especially repeated exposure—can elicit anti-HLA responses, which may affect safety and possibly efficacy, and should therefore be monitored and systematically reported. The ASD phase I program illustrates that *de novo* anti-HLA antibodies can occur despite short-term clinical tolerability (Sun et al., 2020). Beyond alloimmunity, safety assessment must explicitly address tumorigenicity (including long-term surveillance for ectopic tissue formation), thromboembolic/vascular events (particularly for intra-arterial delivery), infection risks (procedural or immunomodulatory), and careful attribution of adverse events to product versus procedure. Importantly, long-term efficacy and follow-up data remain limited across many indications, and durable disability endpoints often require multi-year observation to distinguish transient symptomatic effects from disease modification.

Patient selection should be tightened not only to reduce variance but also to align with clinical equipoise and meaningful unmet need. In CP, where developmental trajectories and rehabilitation response vary widely, RCTs that paired standardized rehabilitation with MSC administration and used GMFM-based outcomes showed larger gains in motor and comprehensive function than placebo over months, emphasizing that rehabilitation intensity and baseline functional reserve must be harmonized across arms (Gu et al., 2020; Huang et al., 2018).

For Parkinson’s disease, early phase MSC studies have generally enrolled idiopathic PD and emphasized safety (Schiess et al., 2021). As the field moves toward efficacy-testing, inclusion criteria should prioritize more homogeneous and clinically meaningful populations. One pragmatic strategy is to enroll patients with advanced disease who have exhausted standard options—such as individuals who failed after deep brain stimulation (DBS) or those who are not candidates for DBS—because the expected trajectory is more predictable and the therapeutic window can be anchored to well-defined clinical milestones. Alternatively, trials could focus on non-idiopathic parkinsonian syndromes (atypical parkinsonism) where the unmet need is greatest; however, these syndromes are themselves heterogeneous and often overlap mechanistically with disorders like MSA or PSP, necessitating stringent diagnostic and biomarker criteria to preserve interpretability.

Collectively, the updated evidence map suggests that MSC therapies are best viewed as platform biologics whose clinical signal—if present—will depend on matching product properties and delivery route to disease mechanism, selecting participants with comparable biology and time-course, and adopting endpoints that reflect plausible MSC effects (immunomodulation, neurovascular stabilization, and circuit-level recovery). The next generation of trials should therefore combine rigorous randomization/blinding where feasible, standardized concomitant care, transparent adverse-event attribution, and harmonized manufacturing/potency testing to enable cross-study comparability and meta-analytic synthesis.

## Limitations

This review is limited by substantial variability across MSC studies, including heterogeneity in protocols, cell sources, dosing regimens, delivery routes, and patient populations, which constrains direct comparability and precludes simple cross-indication generalization. In addition, long-term efficacy evidence is limited: many studies emphasize short-term feasibility and safety, and only a minority include multi-year outcomes or robust disability endpoints. Product-level limitations include incomplete reporting of manufacturing variables (passage number, post-thaw viability, potency assays), variability in GMP workflows across sites, and under-reporting of standardized concomitant rehabilitation and medication adjustments. Finally, incomplete public access to full texts and Supplementary Tables for some trials limited the extent of numeric extraction; where baseline-to-follow-up score tables or AE breakdowns were not directly accessible, we explicitly labeled those elements as not reported/not accessible in Supplementary Table S1.

## Future directions

Priority directions for future work include harmonized potency and release criteria linked to mechanism, standardized reporting of manufacturing variables (donor characteristics, passage, cryopreservation, post-thaw viability), and large-scale GMP workflows capable of producing consistent lots across centers. Future trials should incorporate route-specific risk/benefit frameworks with explicit attention to BBB penetration and biodistribution, and should measure target engagement using biomarkers and imaging, where feasible. Given likely limited MSC persistence, dosing schedules should be optimized (including repeat dosing where transient effects are expected) and aligned with pharmacodynamic readouts. Immune compatibility should be monitored systematically (anti-HLA responses, donor-specific antibodies), particularly for repeated allogeneic regimens. Safety programs should include long-term surveillance for tumorigenicity/ectopic tissue formation, vascular complications, and rigorous adverse-event attribution. Finally, patient selection and responder enrichment should be central design elements, prioritizing homogeneous cohorts, prespecified stratification, and endpoints aligned with meaningful disability trajectories over multi-year follow-up timepoints.
